# Phosphate Deficiency: A Tale from the End of PILNCR2

**DOI:** 10.3390/ncrna9040040

**Published:** 2023-07-25

**Authors:** Santosh Kumar Upadhyay

**Affiliations:** Department of Botany, Panjab University, Chandigarh 160014, India; skupadhyay@pu.ac.in

**Keywords:** miRNA399, phosphate, *PHT1s*, *PLNCR2*, Pi-deficient conditions

## Abstract

A deficiency in inorganic phosphate (Pi) induces the expression of miRNA399 and the accumulation of its target Pi transporters (*PHT1s*) mRNA, which is contrary to the goal of miRNA-mediated gene regulation. Recently, a novel mechanism of RNA/RNA-duplex formation between the transcripts of a Pi deficiency-induced long non-coding RNA (*PILNCR2*) and *PHT1s* has been reported, which prevents the binding and cleavage of miRNA399 to *PHT1* mRNAs, thereby providing tolerance of Pi-deficient conditions. Moreover, the way in which ribosomes move through the RNA/RNA-duplex for the translation of PHT1 transporter proteins remains elusive.

## 1. Introduction

Plants obtain phosphorus (P) as inorganic phosphate (Pi) from the soil to enable their growth. However, the largest proportion of agricultural areas shows Pi deficiency due to its slow diffusion, which restrains crop productivity [1]. Various Pi transporters (PHTs) and transcription factors, like Phosphate Starvation Response (PHRs) and Sensitive to Proton Rhizotoxicity 1 (STOP1), are involved in the transportation and regulation of Pi in plants [2,3,4]. An ubiquitin-conjugating E2 enzyme (PHO2) inhibits PHTs and phosphate 1 (PHO1) transporters to reduce Pi uptake. In contrast, miR399, after inducing expression during conditions of Pi deficiency, suppresses *PHO2* expression during Pi homeostasis [5]. In addition, Du et al. [5] showed that Pi deficiency-induced lncRNA (*PILNCR1*) could protect *ZmPHO2* mRNAs by inhibiting ZmmiRNA399. Moreover, miRNA399 has also been assumed to target *PHT* genes in maize [6], which suggests that additional investigation is required to establish the mechanism of Pi homeostasis, particularly in maize. Recently, Wang et al. [7] revealed the lncRNA (*PILNCR2*)-mediated regulation of interactions between miRNA399 and *PHT1* genes during Pi homeostasis in maize.

## 2. miRNA399 Targets *PHT1* Family Genes

The *PHT1* is a multigene family in maize that comprises 13 members, of which five genes (*PHT1;1*, *1;3*, *1;7*, *1;8*, and *1;13*) have been predicted as potential miRNA399 targets [6,8]. Since Pi deficiency induces ZmmiRNA399 expression, it is believed that the target genes should be downregulated. Therefore, Wang et al. [7] first analysed the expression of target *ZmPHT1* genes in the root tissues using strand-specific RNA libraries generated in Pi-deficient conditions from two inbred lines (CCM454 and 31778) [9] and further validated via quantitative real-time PCR (qRT-PCR). Contrary to our expectations, the results revealed upregulated expression of three *ZmPHT1* genes (*ZmPHT1;1, 1;3,* and *1;13*) under conditions of Pi deficiency, which raised concerns about the validity of targeting *ZmPHT1* genes by ZmmiRNA399. Therefore, this study then validated the interaction between ZmmiRNA399 and mRNAs of *ZmPHT1* genes. The transient co-expression assays revealed reduced accumulation of *ZmPHT1* transcripts in the presence of miRNA399b in *Nicotiana benthamiana*. In addition, the reduced expression of *ZmPHT1;1*, *1;3*, and *1;13* genes was reported in the transgenic maize that overexpress the *MIRNA399b* [5]. Further, these *ZmPHT1* genes comprised potential cleavage sites for miRNA399. These results assured that the mRNAs of *ZmPHT1* genes are potential targets for miRNA399. Moreover, the Pi deficiency-induced upregulated expression of both miRNA399 and its target *ZmPHT1* genes indicates the existence of an alternate regulatory pathway/mechanism.

## 3. *PILNCR2* Regulates miRNA399 Interaction with *ZmPHT1* Genes

In an earlier study, *PILNCR1*-mediated regulation of Pi homeostasis was reported, in which *PILNCR1* was shown to protect *ZmPHO2* mRNAs by inhibiting their cleavage by ZmmiRNA399 [5]. Therefore, Wang et al. [7] explored the lncRNA libraries of CCM454 and 31,778 inbred lines of maize generated under conditions of Pi deficiency [5], and identified *PILNCR2* as a *cis*-natural antisense transcript (NAT) that has a complementary sequence to *ZmPHA1;1*. Analysis of full-length cDNA sequences obtained via RACE and calf intestine alkaline phosphatase (CIP) treatment suggested the occurrence of 5’-capping and 3’-poly-A tails in *PILNCR2*, indicating its RNA polymerase II-mediated transcription. Further, none of the ORFs predicted in the *PILNCR2* sequence could encode functional protein, which confirmed that it is a lncRNA. The expression profiling suggested the upregulation of *PILNCR2* under Pi deficiency, and northern blotting confirmed its localisation in the cytoplasm. The in situ hybridisation indicated the co-occurrence of *PILNCR2* and *ZmPHT1;1*, *1;3*, and *1;13* transcripts throughout the root. These findings suggested the coregulation of *PILNCR2* and *ZmPHT1s* via an elusive mechanism.

**Figure 1:**
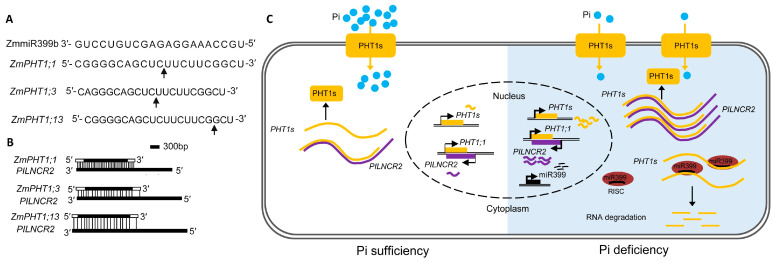
*PLNCR2*-mediated regulation of Pi deficiency. (**A**) shows the ZmmiRNA399 and its target site (indicated by arrows) in *ZmPHT1* mRNAs. (**B**) RNA/RNA duplex formed between the transcripts of *PLNCR2* and *ZmPHT1* genes, which inhibit binding of ZmmiRNA399 to *ZmPHT1* mRNAs. (**C**) A model that demonstrates the normal expression of *PHT1* genes and *PLNCR2* during conditions of Pi sufficiency, while Pi deficiency induces the expression of *PHT1* genes and ZmmiRNA399, along with the *PLNCR2* from the opposite strand of *PHT1;1.* All transcripts translocate to the cytoplasm, where the *PLNCR2* transcripts form RNA/RNA duplexes with the mRNAs of *PHT1s* due to their complementary sequences. The RNA duplex prevents the binding of ZmmiRNA399 to mRNAs of *PHT1s*, protecting them from cleavage. These results highlight the increased accumulation of *PHT1* transcripts that form more PHT1s used for the transportation of Pi into the cytoplasm, thereby proving low Pi tolerance. The figure is adapted and modified from the study of Wang et al. [7], with permission given by Elsevier through Rightslink, licence number 5580761357750.

The *cis*-NATs usually form dsRNA or siRNAs from the overlapping region (OR) of a gene pair to regulate gene expression [10]. Moreover, the current study could not recognize siRNA that originated from *PILNCR2* or ZmPHT1;1 ORs. The RNA–RNA binding assays performed in in vitro conditions using BrU-labelled *PILNCR2* RNA and anti-BrdU mAb antibodies revealed higher accumulations of *ZmPHT1;1*, *1;3*, and *1;13* transcripts when incubated with overlapping *PILNCR2*, thereby suggesting the formation of a RNA/RNA duplex between *PILNCR2* and *ZmPHT1* transcripts. The in vivo ribonuclease protection assay confirmed the above interaction. Additionally, the co-expression analyses of ZmMIR399b combined with *ZmPHT1;1*, *1;3*, or *1;13* in the presence and absence of *PILNCR2* were performed for *N. benthamiana*. The qRT-PCR analyses showed that higher accumulations of mRNAs of *ZmPHT1;1, 1;3*, or *1;13* were present for *PILNCR2*, suggesting that they were protected from ZmMIR399b-mediated cleavage. Even the mutations or deletions in ZmmiR399-overlapping regions in *PILNCR2* were not able to inhibit duplex formation. The RNA/RNA duplex, which was generated between the transcripts of *PILNCR2* and *ZmPHT1s,* interferes with the binding of ZmmiRNA399 to the mRNAs of the *ZmPHT1* family of genes, ultimately protecting them from miRNA-mediated cleavage (Figure 1).

## 4. *PILNCR2* Expression Promotes Low-Pi Tolerance

Since *PILNCR2* protects *ZmPHT1s* from miRNA-mediated degradation and showed inducible expression under Pi-deficient conditions, one questions remain to be answered: is it involved in low-Pi tolerance? To answer this question, Wang et al. [7] generated overexpression (OE), knock-down (KD), and knock-out (KO) lines for *PILNCR2* in the maize plants and analysed them for low Pi tolerance. The *PILNCR2* OE provided significant tolerance in the maize plants under low-Pi conditions without accumulation of anthocyanin. However, both KO and KD lines showed higher accumulation of anthocyanin under low-Pi treatment than the wild type and OE lines. The Pi-depletion assay revealed a higher rate of Pi absorption by OE lines and increased accumulation of Pi in various plant parts. In contrast, KO and KD lines exhibited highly reduced Pi absorption rates and depleted P accumulation in roots and shoots, even in the Pi-sufficient condition. Moreover, *PILNCR2* KO abolished Pi-toxicity caused by ZmmiRNA399b OE in the double transgenic maize under Pi-sufficient conditions. Du et al. [5] earlier reported that the OE of ZmMIRNA399b caused Pi toxicity by downregulating the expression of *ZmPHO2*. Additionally, Wang et al. [7] found that the expression level of ZmMIRNA399 could not be affected by both OE and KO of *PILNCR2*, though *ZmPHT1* genes showed increased expression in OE and decreased expression in KO lines of *PILNCR2* transgenic maize. Further, the ZmMIRNA399 OE did not affect *PILNCR2* expression, though it reduced the accumulation of transcripts of *ZmPHT1* genes. These results suggest the function of *PILNCR2* in conditions of low Pi tolerance as being to protect the mRNAs of *ZmPHT1* genes from ZmmiRNA399-mediated degradation.

## 5. Concluding Remarks and Future Perspective

In summary, *PILNCR2* regulates Pi homeostasis in maize, and its induced expression provides low Pi tolerance by protecting the ZmmiRNA399-mediated cleavage of *PHT1* mRNAs. The Pi deficiency-induced upregulation of both ZmmiRNA399 and its target *PHT1* genes suggested an alternative mechanism of regulation. The *PILNCR2* transcribed from the opposite strand of *ZmPHT1;1* forms RNA/RNA duplex in combination with the mRNAs of *ZmPHT1* genes, preventing binding of ZmmiRNA399. The study provides a novel mechanism for lncRNA-mediated regulation of gene expression. Moreover, the study raises a few interesting questions. Since the transcript of *PILNCR2* completely masked the mRNAs of *ZmPHT1s* from the 5′ end to the 3′ end, how did ribosomes bind and move through the RNA/RNA duplex during translation? The mechanism of ribosome movement through RNA/RNA duplex would reveal a novel avenue for the functioning of lncRNAs, especially the NATs. Further, the accumulation of ZmPHT1 proteins could be analysed in tandem with their transcript abundance. This observation raises another question: is this method the only way to determine low Pi tolerance in plants? Alternatively, there may be some other transporters or regulatory mechanism available that function in coordination with this pathway; this possibility needs to be addressed in future studies.

## Data Availability

Data sharing not applicable. No new data were created in this study.
